# Precise Control of Water Adsorption Behavior in Solid Solutions of Amorphous Coordination Polymers

**DOI:** 10.1002/chem.71070

**Published:** 2026-05-08

**Authors:** Haruka Yoshino, Kohei Yamagami, Yuki Imamura, Hirona Yamagishi, Sayuri Shimoda, Benjamin Le Ouay, Ryo Ohtani, Hitoshi Miyasaka, Masaaki Ohba

**Affiliations:** ^1^ Department of Chemistry Faculty of Science Kyushu University Fukuoka Japan; ^2^ Institute For Materials Research Tohoku University Sendai Japan; ^3^ Japan Synchrotron Radiation Research Institute Sayo‐cho Hyogo Japan; ^4^ Synchrotron Radiation Center Ritsumeikan University Kusatsu Shiga Japan

**Keywords:** amorphous, coordination polymers/ metal–organic frameworks, solid solutions, water adsorption, X‐ray absorption fine structure (XAFS)

## Abstract

Metal‐organic frameworks (MOFs) and coordination polymers (CPs) with flexible coordination networks can exhibit rapid adsorption accompanied by structural changes at a specific pressure called the gate‐opening pressure (*P*_go_), making them well‐suited for various water‐uptake‐based applications. Herein, we demonstrated the precise modulation of the water uptake behavior in the amorphous coordination polymers (**Am‐CPs**), Co*_x_* M*_(1‐x)_* [Ni(CN)_4_] (**Co*_x_* M*_(__1__‐x)_***; M = Mn, Fe), via a solid solution approach. All amorphous solid solutions of **Co*_x_* M*_(__1__‐x)_*** showed abrupt water adsorption within the relative pressure range of *P*/P_0_ = 0.5–0.9. This phenomenon was associated with an amorphous–to–crystalline transformation into the 2D Hofmann‐type analog of Co*_x_* M*_(1‐x)_* (H_2_O)_2_[Ni(CN)_4_]·4H_2_O (**Co*_x_* M*_(__1__‐x)_*‐H_2_O**). Systematic changes in *P*_go_ for **Co*_x_* M*_(__1__‐x)_*** were controlled by the type and ratio of the central metals, which were revealed using a combination of *L*_2,3_‐ and *K*‐edge X‐ray absorption fine structure (XAFS) techniques. Slight differences in the bond energies of bonds in the vicinity of the water‐induced amorphous–to–crystalline transformation. This transformation involves a change in the coordination geometry, from tetrahedral to octahedral, through the rearrangement of CN‐Co(M) bonds via cleavage/reformation, giving rise to the desirable water uptake characteristics.

## Graphical abstract

Amorphous coordination polymers (**Am‐CPs**) showed a strict modulation of water uptake behavior via a solid solution approach. The desirable water uptake characteristics were corroborated by the type and local coordination geometry of the central metals, as revealed by a combination of *L*_2,3_‐ and *K*‐edge X‐ray absorption fine structure (XAFS) techniques.

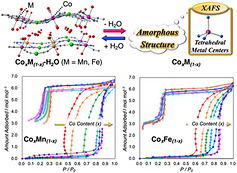

## 1 Introduction

Metal‐organic frameworks (MOFs) and coordination polymers (CPs) comprised of metal ions and organic ligands have garnered attention as functional materials because of their suitability for applications in gas storage [1, 2, 3], separation [4, 5, 6], sensing [7, 8, 9, 10], and catalysis [11, 12, 13]. Appropriate fine‐tuning of the functionalities of MOFs/CPs through the modification of structural components of the coordination frameworks is crucial for promoting their applications, and in particular, various strategies have been explored to optimize their guest adsorption properties [14, 15, 16, 17, 18, 19, 20]. Notably, the use of solid solutions of MOFs/CPs obtained by mixing metal ions or ligands is a promising approach for effective manipulation of the guest uptake and release behavior [21, 22, 23, 24, 25]. For example, a series of mixed‐ligand solid solutions of [Zn_2_(bdc)_2‐_ *_a_* (bdc–X)*_a_* (bpy)] (H_2_bdc = 1,4‐benzenedicarboxylic acid; bpy = 4,4’‐bipyridine; X = −NO_2_ or −NH_2_) exhibited favorable acetylene storage performance at room temperature (RT), with the adsorption isotherm could be effectively controlled by the steric and electronic characters of the organic ligands incorporated in the framework [26]. Although the crystalline features of MOFs/CPs enable the precise investigation of the porosity‐related capacity and physical properties, the inherent drawbacks of poor structural robustness, poor processability, and mechanical instability must be addressed to meet the requirements for practical applications. This has motivated an ongoing research effort for investigations of the functionalities of noncrystalline and amorphous MOFs/CPs (**Am‐MOFs/CPs**), which have shown remarkable promise for realizing beneficial characteristics such as enhanced guest adsorption capacity, luminescence behavior, and proton conductivity [27, 28, 29]. These properties are attributed to the more open and dynamic frameworks of **Am‐MOFs/CPs** compared to those of crystalline MOFs/CPs, which are obtained through deliberate amorphization. However, while many studies on the application of solid solutions in crystalline MOFs/CPs have been reported, investigations on the effect of solid solution formation on the functionality of **Am‐MOFs/CPs** have been scarce, owing to the technical difficulties involved in investigations of structure‐related functionalities of amorphous materials [30]. Accordingly, the effects of subtle adjustments of the metal nodes in the amorphous frameworks on the overall adsorption properties of **Am‐MOFs/CPs** remain largely unexplored.

Given the pronounced adsorption behavior of MOFs/CPs at a certain relative pressure along with a small temperature window and a large working capacity (i.e., gate adsorption), their water uptake is expected to be utilized in various water‐related applications [31, 32, 33]. Generally, the gate‐opening pressure (*P*_go_) of MOFs/CPs depends strongly on the pore size and geometry, the flexibility of the coordination networks, and the strength of the host−guest interactions related to the hydrophilicity or hydrophobicity of the internal space [34, 35, 36, 37, 38, 39, 40]. This dependence enables various practical applications, such as desiccation, separation of water from other chemical species, water harvesting, heat pumping/chilling, desalination, humidity control, and dehydration of organic solvents [41, 42, 43, 44, 45, 46, 47, 48, 49, 50, 51, 52, 53, 54, 55]. Additionally, water adsorption on MOFs/CPs often occurs at RT, and the regeneration of the framework requires only a low driving temperature in contrast to the classical sorbents (*ca*. < 85°C) [56]. Despite the promising potential of the application of MOFs/CPs as water adsorbents, materials that meet the requirements for practical applications remain largely unexplored [34, 57, 58]. Thus, a comprehensive evaluation of new categories of materials is essential, and the development of an inexpensive combination of structural components for MOFs/CPs with 3D ions and light atoms is necessary to reduce operational costs and promote their widespread uses.

In this study, we demonstrate the adjustable water adsorption behavior for solid solutions of cyanide‐bridged **Am‐CPs**, Co*_x_* M*_(1‐x)_* [Ni(CN)_4_] (**Co*_x_* M*_(1‐x)_***; M = Mn, Fe), through the cleavage/reformation of CN‐Co(M) bonds. Detailed analyses of the coordination geometries and bond lengths around the Co^2+^ and M^2+^ centers using *L*_2,3_‐ and *K*‐edge X‐ray absorption fine structure (XAFS) spectroscopy revealed that the local structure of the metal nodes gives rise to the water adsorption pressure that drives the macroscopic amorphous–to–crystalline conversions. To the best of our knowledge, the resultant adjustable water uptake property of **Co*_x_* M*_(1‐x)_*** is the first demonstration of controlling the adsorption behavior of **Am‐MOFs/CPs** using a solid solution approach.

## 2 Results and Discussion

**FIGURE 1:**
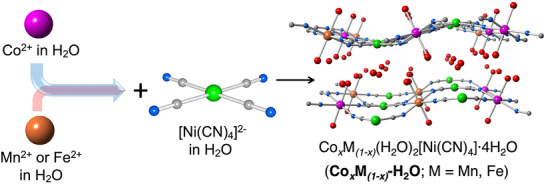
Schematic illustration of the synthesis of mixed‐metal solid solutions Co_x_M_(1‐x)_(H_2_O)_2_[Ni(CN)_4_]·4H_2_O (**Co*_x_* M*_(1‐x)_*‐H_2_O**; M = Mn, Fe) based on crystalline 2D Hofmann‐type CPs.

**FIGURE 2:**
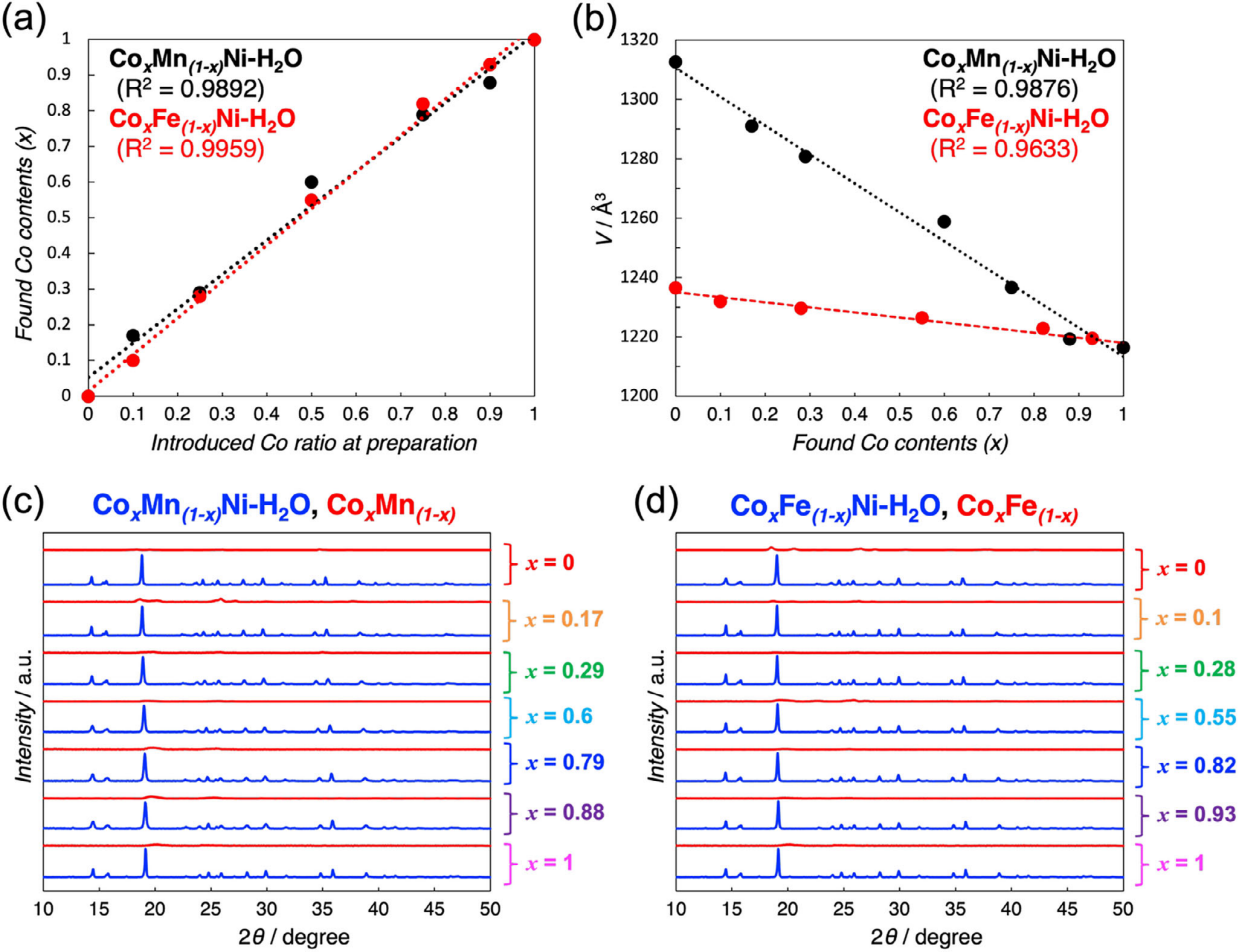
(a) Molar percentage of the found Co^2+^ (*x*) within **Co*_x_* M*_(1‐x)_*‐H_2_O** obtained as a function of the molar percentage of the introduced Co^2+^ in the reactions. (b) Variations of cell volume for **Co*_x_* M*_(1‐x)_*‐H_2_O** at RT, calculated from Le Bail analyses using PXRD patterns. (c), (d) PXRD patterns of **Co*_x_* M*_(1‐x)_*‐H_2_O** (blue) and **Co*_x_* M*_(1‐x)_*** (red).

All samples of the crystalline 2D Hofmann‐type CPs M(H_2_O)_2_[Ni(CN)_4_]·4H_2_O (**MNi‐H_2_O**; M = Mn, Fe, Co) and their solid solutions Co*_x_* M*_(1‐x)_* (H_2_O)_2_[Ni(CN)_4_]·4H_2_O (**Co*_x_* M*_(1‐x)_*‐H_2_O**) were prepared in accordance with previously reported procedures [59, 60]. The procedure for preparation of **Co*_x_* M*_(1‐x)_*‐H_2_O** is illustrated in Figure 1 and is described in detail in the Experimental Section. **MNi‐H_2_O** formed undulating 2D layer structures consisting of [Ni(CN)_4_]^2−^ and M^2+^, wherein two water molecules occupied the axial positions of the M^2+^ centers as co‐ligands and the other four molecules were located between the layers. Metal‐based solid solutions (**Co*_x_* M*_(1‐x)_*‐H_2_O**) with different Co^2+^ contents (*x*) were systematically synthesized using a procedure similar to that used for the synthesis of **MNi‐H_2_O**, except that a mixture of metal ions was used. X‐ray fluorescence analysis of **Co*_x_* M*_(1‐x)_*‐H_2_O** indicated that the Co^2+^ molar content (*x*) in the framework was proportional to the ratio of Co^2+^ introduced during preparation (Figure 2a), enabling convenient modulation of *x* for **Co*_x_* Mn*_(1‐x)_*‐H_2_O** and **Co*_x_* Fe*_(1‐x)_*‐H_2_O** in the range from 0 to 1 (Tables S1, S2). In addition, the homogeneous distribution of Co^2+^, Mn^2+^, and Fe^2+^ within the structures was confirmed by scanning electron microscopy with energy‐dispersive X‐ray (SEM‐EDX) spectroscopy (Figure S1). The powder X‐ray diffraction (PXRD) patterns of **Co*_x_* M*_(1‐x)_*‐H_2_O** were similar to those of **MNi‐H_2_O** with systematic and continuous peak shifts (Figure S2), corresponding to differences between the ionic radii of the central metals (Mn^2+^, Fe^2+^, and Co^2+^) in the framework. Moreover, Le Bail analyses of **MNi‐H_2_O** and **Co*_x_* M*_(1‐x)_*‐H_2_O** were performed using the FOX and RIETAN‐FP software [61, 62], and showed that the changes in the cell parameters conformed to Vegard's law (Figures 2b and S3–S5; Tables S3, S4) [29, 63]. The Fourier transform infrared (FT‐IR) spectrum of **Co*_x_* M*_(1‐x)_*‐H_2_O** shown in Figure S6 depict the O─H stretching and H─O─H bending modes assigned to water molecules at approximately 1600 and 3000–3600 cm^−1^, respectively. Furthermore, the gradual shift of the bridging CN^−^ moieties at approximately 2160 cm^−1^ showed good agreement with the trends of the Le Bail analyses, demonstrating the formation of single‐phase mixed‐metal solid solutions.

**FIGURE 3:**
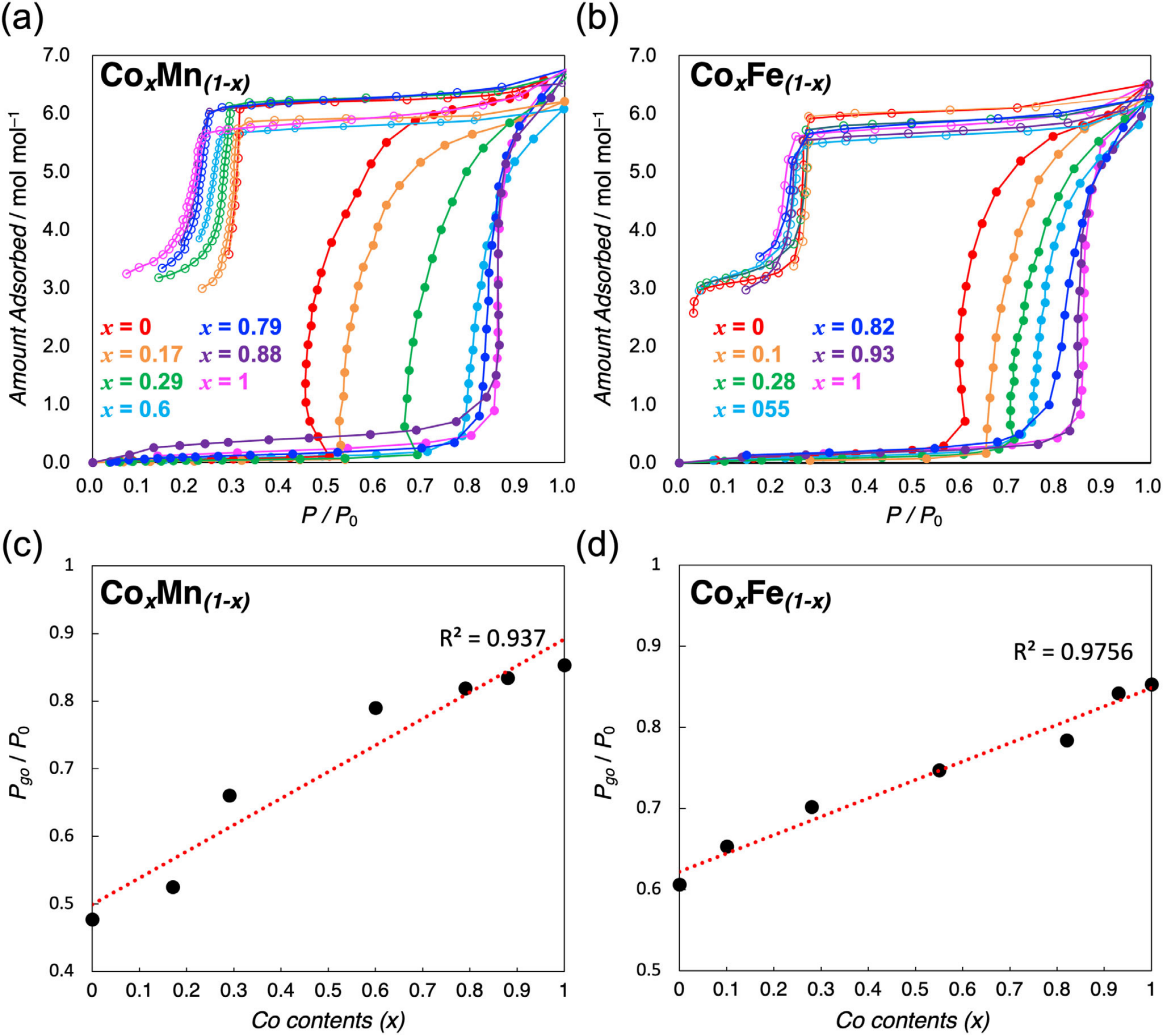
(a) H_2_O adsorption/desorption isotherms of (a) **Co*_x_* Mn*_(1‐x)_*** and (b) **Co*_x_* Fe*_(1‐x)_*** at 298 K. The filled and open circles represent the adsorption and desorption processes, respectively. *P*_go_ vs. Co contents (*x*) plots of (c) **Co*_x_* Mn*_(1‐x)_*** and (d) **Co*_x_* Fe*_(1‐x)_*** obtained from water adsorption/desorption data at 298 K.

In situ PXRD patterns were measured under vacuum to investigate the structural changes in **Co*_x_* M*_(1‐x)_*‐H_2_O** during dehydration (Figure 2c, d). Dehydrated compounds (**Co*_x_* M*_(1‐x)_***) were obtained by heating **Co*_x_* M*_(1‐x)_*‐H_2_O** at 353 K under vacuum, thereby resulting in crystalline–amorphous transformations for all solid solution compounds. Although thermal treatment led to the formation of low‐crystallinity materials via water removal, the FT‐IR spectra revealed that the cyanide‐bridged networks with N‐coordinated M^2+^ centers maintained their integrity (Figure 5e, f). Water‐induced structural changes in **Co*_x_* M*_(1‐x)_*** were confirmed by PXRD analysis. The rehydrated **Co*_x_* M*_(1‐x)_*** (**Co*_x_* M*_(1‐x)_*‐H_2_O** (rehydration)) was obtained via the vapor diffusion method, as indicated by the PXRD results which showed reversible interconversion of amorphous **Co*_x_* M*_(1‐x)_*** into crystalline **Co*_x_* M*_(1‐x)_*‐H_2_O** (rehydration; Figure S7–9). Notably, the crystallinity of the rehydrated **Co*_x_* M*_(1‐x)_*‐H_2_O** (rehydration) was maintained even after one month under vapor‐diffusion conditions, indicating the favorable structural stability of **Co*_x_* M*_(1‐x)_*** in a high‐humidity environment. Water adsorption/desorption measurements were performed to investigate the water uptake of **Co*_x_* M*_(1‐x)_***. It was found that all **Co*_x_* M*_(1‐x)_*** exhibited abrupt water adsorption behaviors with approximately six water molecules adsorbed per unit at 298 K (Figure 3a, b); however, the *P*_go_ value was strongly influenced by the ratio of the metal ions in **Co*_x_* M*_(1‐x)_***: the range of *P*_go_ of **Co*_x_* Mn*_(1‐x)_*** and **Co*_x_* Fe*_(1‐x)_*** were 0.48< *P*_go_ < 0.85 and 0.61< *P*_go_ < 0.85, respectively. A good correlation was observed between *P*_go_ and the Co^2+^ content (*x*) of **Co*_x_* M*_(1‐x)_*** over the entire range (Figure 3c, d). Thus, the water uptake properties of **Co*_x_* M*_(1‐x)_*** were tuned by slight changes in the composition of the metal center of the framework. To the best of our knowledge, the observed tuning of the water uptake properties of **Co*_x_* M*_(1‐x)_*** represents the first demonstration of the control of the adsorption behavior of **Am‐MOFs/CPs** via a solid‐solution approach.

**FIGURE 4:**
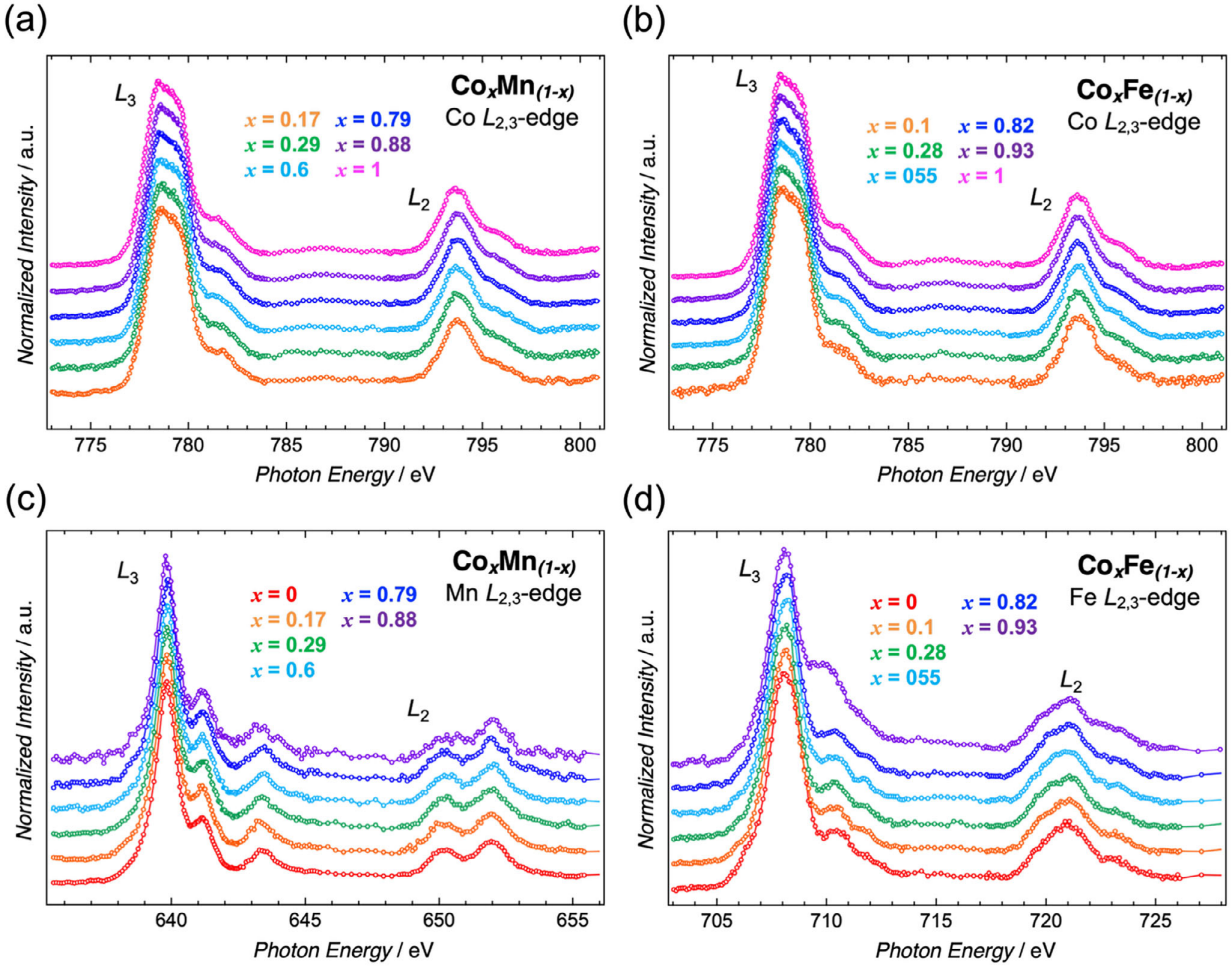
(a) Co *L*_2,3_‐edge XAFS spectra of (a) **Co*_x_* Mn*_(1‐x)_*** and (b) **Co*_x_* Fe*_(1‐x)_***. (c) Mn *L*_2,3_‐edge XAFS spectra of **Co*_x_* Mn*_(1‐x)_***. (d) Fe *L*_2,3_‐edge XAFS spectra of **Co*_x_* Fe*_(1‐x)_***.

To gain insights into the influence of the metal ion species in the amorphous **Co*_x_* M*_(1‐x)_*** on the *P*_go_ value, the local geometries and bond lengths around the three metal species (Mn^2+^, Fe^2+^, and Co^2+^) were investigated by *L*_2,3_‐ and *K*‐edge X‐ray absorption fine structure (XAFS) techniques. Detailed structural characteristics of **MNi** revealed using experimental and computational approaches were previously reported by our group [60, 64]. Based on the results for **MNi**, we established the origin of the structural effects of the M^2+^ incorporation on the tunable water adsorption behavior of **Co*_x_* M*_(1‐x)_***. The Co *L*_2,3_‐edge XAFS spectra originating from the Coulombic attraction between the 2*p* core‐holes and the 3*d* valence electrons *via* the 2*p*→3*d* absorption process [65, 66] are presented in Figure 4a, b. The notable double‐peak structures of the *L*_3_ (*hv* ≈ 780 eV) and *L*_2_ (*hv* ≈ 796 eV) edges corresponding to the Co 2*p* core‐hole spin‐orbit coupling exhibit a profile similar to those of YBaCo_3_AlO_7_ and **CoNi** with tetrahedral Co^2+^ structure (Figure S11a) [67], indicating that the Co^2+^ in **Co*_x_* M*_(1‐x)_*** was also found in a tetrahedral geometry. Moreover, the Mn and Fe *L*_2,3_‐edge XAFS spectra presented in Figure 4c, d displayed the main peaks assigned to *L*_3_ (*hv* ≈ 641 eV for Mn^2+^, *hv* ≈ 710 eV for Fe^2+^) and *L*_2_ (*hv* ≈ 651 eV for Mn^2+^, *hv* ≈ 722 eV for Fe^2+^), indicating that Mn and Fe local environments showed tetrahedral symmetry that was previously observed in (Zn,Mn)Se, **MnNi**, CsFe_2_Se_3_, and **FeNi** (Figure S11b,c) [68, 69]. Hence, the octahedral coordination of metal ions (Mn^2+^, Fe^2+^, and Co^2+^) of **Co*_x_* M*_(1‐x)_*‐H_2_O** was transformed to tetrahedral coordination during the crystalline‐amorphous transformations via dehydration.

**FIGURE 5:**
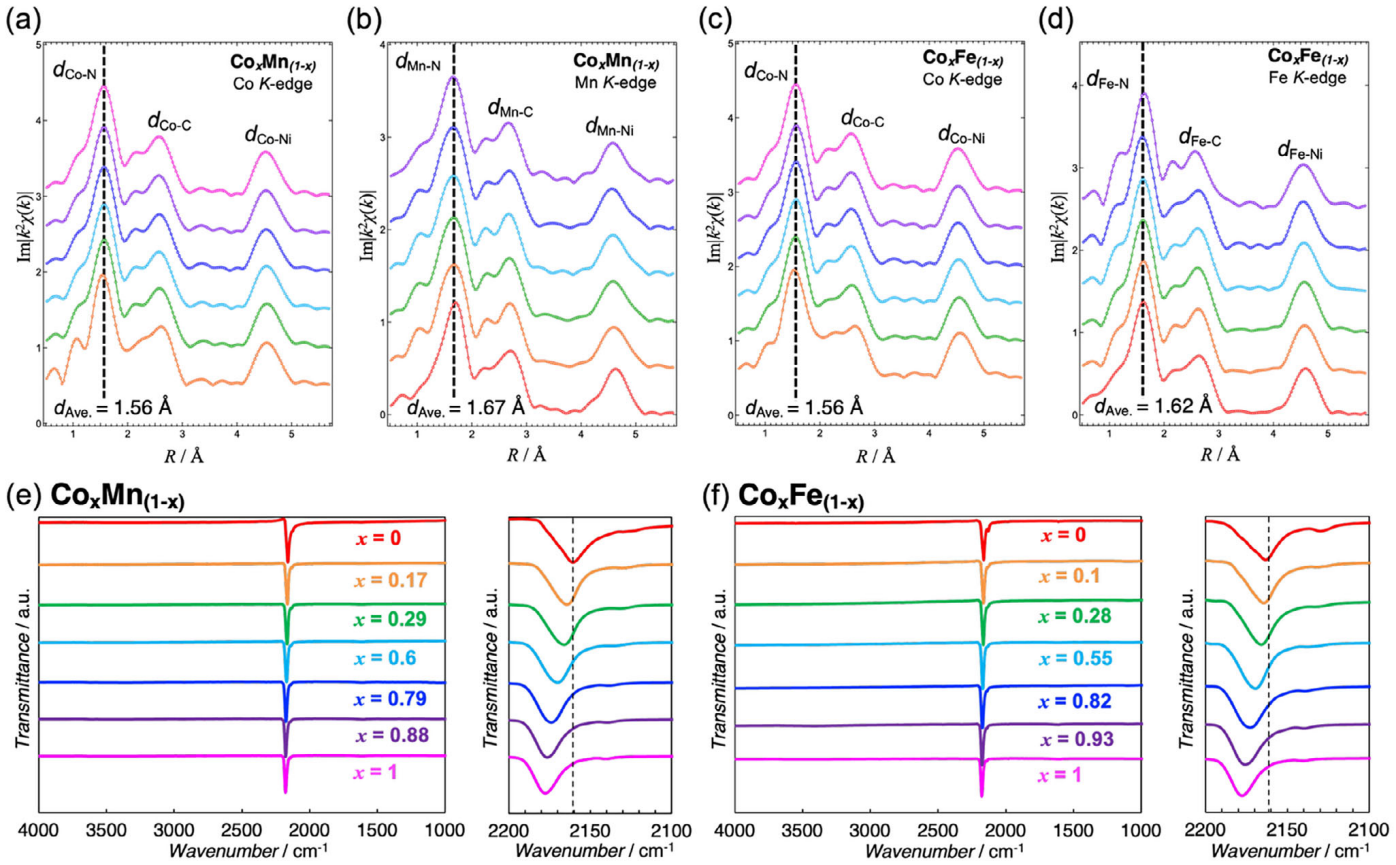
(a)–(d) Fourier‐transformed spectra of **Co*_x_* M*_(1‐x)_*** from the *K*‐edge XAFS measurements. IR spectra of (e) **Co*_x_* Mn*_(1‐x)_*** and (f) **Co*_x_* Fe*_(1‐x)_*‐H_2_O**.

The intrinsic structural features pertaining to the bond lengths around the metal centers for **Co*_x_* M*_(1‐x)_*** were also evaluated through the *K*‐edge XAFS spectra derived from the 1*s*→4*p* excitation process [70, 71, 72, 73] to gain deeper insights into the resultant water adsorption natures. The pre‐edge peaks of **Co*_x_* M*_(1‐x)_*** at approximately 6537 (Mn^2+^ for **Co*_x_* Mn*_(1‐x)_***), 7110 (Fe^2+^ for **Co*_x_* Fe*_(1‐x)_***), and 7707 eV (Co^2+^ for **Co*_x_* M*_(1‐x)_***) in the X‐ray absorption near‐edge structure (XANES) spectra suggest a tetrahedral structure without inversion symmetry (Figure S13), which was consistent with the *L*_2,3_‐edge XAFS spectra. Moreover, a detailed examination of the bond lengths around the central metal atoms was performed using high‐quality EXAFS data. The Fourier‐transformed spectra exhibited the characteristic triple peaks corresponding to the distances between the metal ions (Mn^2+^, Fe^2+^, and Co^2+^) and the neighboring N (*d*_M‐N_), C (*d*_M‐C_), and Ni (*d*_M‐Ni_) atoms of [Ni(CN)_4_]^2‒^ (Figure 5a, d), similar to those previously reported for cyanide‐bridged compounds involving Hofmann‐type MOFs/CPs [60, 74]. The average *d*_M‐N_ values were determined to be 1.67 (*d*_Mn‐N_ for **Co*_x_* Mn*_(1‐x)_***), 1.62 (*d*_Fe‐N_ for **Co*_x_* Fe*_(1‐x)_***), and 1.56 Å (*d*_Co‐N_ for **Co*_x_* M*_(1‐x)_***), respectively (Tables S5,S6). The trend observed for the *d*_M‐N_ values was consistent with a systematic shift of the stretching mode of the CN^−^ groups in the IR spectra of **Co*_x_* M*_(1‐x)_*** (Figures 5e, f).

**FIGURE 6:**
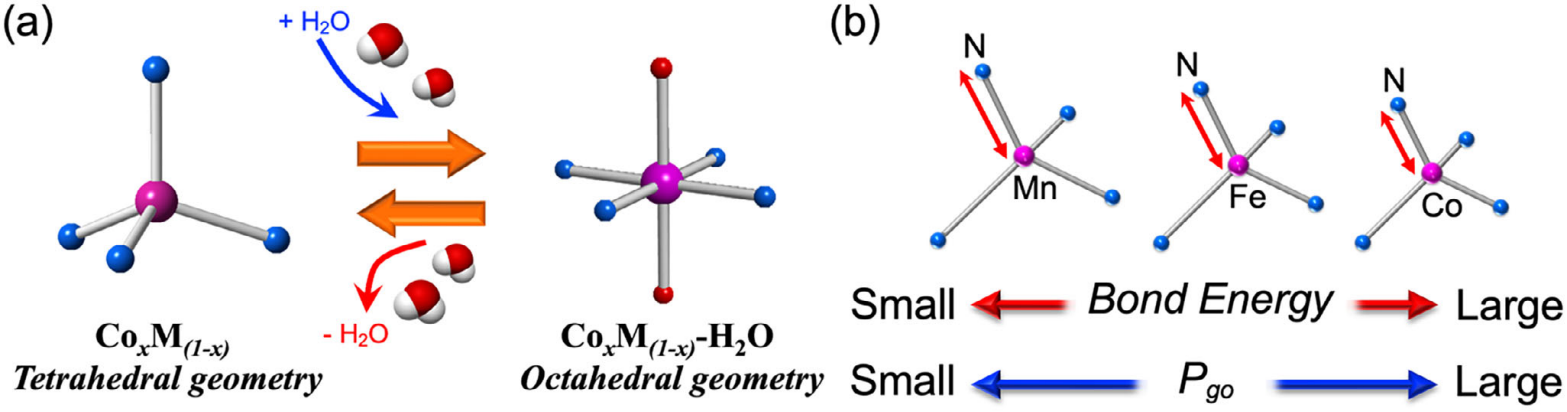
Schematic illustration of (a) change in the coordination geometry between tetrahedral and octahedral geometries for **Co*_x_* M*_(1‐x)_*** during water adsorption/desorption processes. (b) Proposed relationships between the bond energy of M–N bond energy and *P*_go_ values for **Co*_x_* M*_(1‐x)_***.

Finally, we investigated the structural origin of the variation in the *P*_go_ values observed in Figure 3, including the local coordination environment of the M^2+^ ions. All **Co*_x_* M*_(1‐x)_*** with tetrahedral metal centers displayed a steep one‐step water uptake behavior driven by amorphous–to–crystalline transformations (Figure 6a). Notably, a similar adsorption profile has also been reported for Fe[Pd(CN)_4_] with a 3D PtS‐type structure [75]. In the case of Fe[Pd(CN)_4_], the dehydrated sample was not amorphous because heavy Pd centers were periodically incorporated in its framework. The transformation from an anhydrous 3D PtS‐type network to hydrated 2D layers was induced by water adsorption. The adsorption of water onto the Fe sites gave rise to the rearrangement of the cyanide bridges via cleavage and reformation, wherein the ensuing accommodation of the lattice water was observed as the resultant one‐step adsorption driven by the guest‐guest interactions, regardless of the equilibrium time (i.e., hydrogen bonding networks) [75]. The water adsorption behavior depended on the local coordination geometry of the metal ions in **Am‐CPs**. The control material Ni[Ni(CN)_4_] (**NiNi**) with a square‐planar Ni^2+^ geometry after water decoordination showed a two‐step adsorption behavior [76]. The first water adsorption led to an intermediate state of Ni(H_2_O)_2_[Ni(CN)_4_]·H_2_O, and then additional water molecules were accommodated as lattice solvents in the second adsorption. Hence, the local structure of M^2+^ in **MNi** and **Co*_x_* M*_(1‐x)_*** influenced their water adsorption properties and the formation of intermediates. In contrast to the adsorption process, the desorption isotherms showed a plateau in the low‐pressure region (*P*/P_0_< 0.3) for all compounds, which was caused by the formation of intermediate states (M(H_2_O)_2_[Ni(CN)_4_]·H_2_O or Co*_x_* M*_(1‐x)_* (H_2_O)_2_[Ni(CN)_4_]·H_2_O) [59, 76]. The overall water adsorption/desorption behaviors with a hysteresis loop indicate a relatively strong host‐guest interaction [36, 60, 75]. We note that the desorption equilibrium could not be reached within a practical time; however, heating and vacuum treatment at 358 K could easily return the materials to their dehydrated forms (**MNi** or **Co*_x_* M*_(1‐x)_***). These results are characteristic of flexible cyanide‐bridged frameworks, in which structural transformations, including cleavage/reformation of the cyanide bridge, are directly related to the gate‐opening behavior. Importantly, the *P*_go_ values of **Co*_x_* M*_(1‐x)_*** were governed by the type and ratio of the central metal ions, where *P*_go_ increased with decreasing *d*_M‐N_ (*d*_Mn‐N_ > *d*_Fe‐N_ > *d*_Co‐N_). Thus, the *d*_M‐N_ value, which is related to the bond energy, acts as a control variable for *P*_go_. For the smaller *d*_M‐N_ represented by Co^2+^, a higher activation energy (and thus *P*_go_) was required for the amorphous–to–crystalline transitions compared to Mn^2+^ and Fe^2+^ which have larger *d*_M‐N_ values because of the change in the coordination geometry from tetrahedral to octahedral (Figure 6b). Thus, the exceptional combination of drastic structural conversion, such as the amorphous–to–crystalline transformation and dimensional changes demonstrates the potential of **Co*_x_* M*_(1‐x)_*** for realizing tunable steep water adsorption.

## 3 Conclusion

We demonstrated the precise modulation of the water uptake properties in a series of solid solutions based on amorphous CPs (**Am‐CPs**) of Co*_x_* M*_(1‐x)_* [Ni(CN)_4_] (**Co*_x_* M*_(1‐x)_***; M = Mn, Fe). Despite the amorphous nature and lack of long‐range order of these materials, the solid‐solution approach effectively tuned the water uptake pressure while maintaining cooperativity. Amorphous compounds can be easily generated by heating and can be used reversibly. Their excellent water adsorption performance under environmental conditions such as indoor humidity levels makes them suitable for applications in the target relative humidity range between 40% and 65% [77], suggesting that materials beyond crystalline CPs should be examined in adsorption studies. More generally, investigations of the structure‐related functionalities of amorphous materials represent one of the most significant challenges in materials science. The combination of *L*_2,3_‐, *K*‐edge XAFS spectra yielded significant findings regarding local structural features, such as coordination geometries and bond lengths. These findings can facilitate the interpretation of the intrinsic behavior of the amorphous solids. Investigations of the systematic variations in the water adsorption pressure (equivalent to *P*_go_) of the resulting solid solutions caused by the subtle manipulation of the central metals can provide novel insights into the potential applications of amorphous MOFs, such as water‐uptake‐based applications.

## Conflicts of Interest

The authors declare no conflicts of interest.

## Supporting information




**Supporting file 1**: chem71070‐sup‐0001‐SuppMat.docx
