# Supplementary material for: Precise Control of Water Adsorption Behavior in Solid Solutions of Amorphous Coordination Polymers
Source: Chemistry. 2026 May 8;32(31):e71070. doi: 10.1002/chem.71070 (PMC13485169; doi:10.1002/chem.71070)
Supplement: Supplementary file 1 [file CHEM-32-e71070-s001.docx]

Supporting Information

**Precise Control of Water Adsorption Behavior in**

**Solid Solutions of Amorphous Coordination Polymers**

Haruka Yoshino,*^[a,b]^ Kohei Yamagami,^[c]^ Yuki Imamura,^[a]^ Hirona Yamagishi,^[d]^ Sayuri Shimoda,^[a]^ Benjamin Le Ouay,^[a]^ Ryo Ohtani,^[a]^ Hitoshi Miyasaka,^[b]^ and Masaaki Ohba*^[a]^

*^a^ Department of Chemistry, Faculty of Science, Kyushu University, 744 Motooka, Nishi-ku, Fukuoka 819-0395, Japan. E-mail: ohba@chem.kyushu-univ.jp*

*^b^ Institute for Materials Research, Tohoku University, 2-1-1 Katahira, Aoba-ku, Sendai 980-8577, Japan. E-mail: haruka.yoshino.a2@tohoku.ac.jp*

*^c^ Japan Synchrotron Radiation Research Institute, 1-1-1 Kouto, Sayo-cho, Sayo-gun, Hyogo 679-5198, Japan*

*^d^ Synchrotron Radiation Center, Ritsumeikan University, Kusatsu, Shiga 525-0058, Japan*

*Corresponding author:

Dr. Haruka Yoshino

Department of Chemistry, Faculty of Science, Kyushu University, 744 Motooka, Nishi-ku, Fukuoka 819-0395, Japan; Institute for Materials Research, Tohoku University, 2–1–1 Katahira, Aoba-ku, Sendai, Miyagi 980-8577, Japan

E-mail: haruka.yoshino.a2@tohoku.ac.jp

Tel: +81-22-215-2033, FAX: +81-22-215-2031

orcid.org/0000-0003-3102-6222

Prof. Dr. Masaaki Ohba

Department of Chemistry, Faculty of Science, Kyushu University, 744 Motooka, Nishi-ku, Fukuoka 819-0395, Japan

E-mail: ohba@chem.kyushu-univ.jp

Tel, FAX: +81-92-802-4152

orcid.org/ 0000-0001-9268-3512

**Contents for SI**

**Experimental Section**····································································	S3-5

**Fig. S1**. SEM images and elemental mapping ·······································	S6

**Table S1, S2**. Co Contents from XRF data ··········································	S7

**Fig. S2**. PXRD patterns of **Co*_x_*M*_(1-x)_*-H_2_O** ···········································	S8

**Fig. S3-S5**. Le Bail analyses ···························································	S9-S11

**Table S3, S4**. Refined cell parameters of **Co*_x_*M*_(1-x)_*-H_2_O** ·························	S12

**Fig. S6**. IR spectra of **Co*_x_*M*_(1-x)_*-H_2_O** ·················································	S13

**Fig. S7-S9**. *In situ* PXRD patterns under vacuum and water ······················· S14-S16

**Fig. S10**. TGA curves ···································································	S17

**Fig. S11**. *L*_2,3_-edge XAFS spectra of **MNi** ···········································	S18

**Fig. S12**. Co, Mn, and Fe *K*-edge XAFS spectra ····································	S19

**Fig. S13**. *K*-edge XANES spectra of **Co*_x_*M*_(1-x)_*** ·····································	S20

**Table S5, S6**. *d*_M-N_, *d*_M-C_, and *d*_M-N_ values of **Co*_x_*M*_(1-x)_*** ···························	S21

**References in SI** ········································································	S22

**EXPERIMENTAL METHODS**

**Materials**

All chemicals were purchased from commercial sources and used without further purification. M(H_2_O)_2_[Ni(CN)_4_]·4H_2_O (**MNi-H_2_O**; M = Mn, Fe, and Co) species and their solid solutions Co*_x_*M*_(1-x)_*(H_2_O)_2_[Ni(CN)_4_]·4H_2_O (**Co*_x_*M*_(1-x)_*-H_2_O**; M = Mn, Fe) were prepared according to a previously described method.^1,2^

**Synthesis of M(H_2_O)_2_[Ni(CN)_4_]·4H_2_O (MNi-H_2_O; M = Mn, Fe, and Co)**

A solution of K_2_[Ni(CN)_4_]·H_2_O (198.7 mg, 0.767 mmol) in H_2_O (100 mL) was added to a solution of MnCl_2_·4H_2_O (151.8 mg, 0.767 mmol) in H_2_O (80 mL) at RT. White powder samples were collected after stirring for 24 h, washed with water, and air-dried. Yield: 64.5%. **FeNi-H_2_O** was synthesized as a white powder sample in a similar manner to that used for **MnNi-H_2_O**, except for the use of Fe(NH_4_)_2_(SO_4_)_2_·6H_2_O (300.4 mg, 0.766 mmol) in degassed H_2_O (80 mL). Yield: 70.1%. **CoNi-H_2_O** was synthesized as a pink powder sample in a similar manner to that used for **MnNi-H_2_O**, except for the use of CoCl_2_·6H_2_O (180.4 mg, 0.758 mmol). Yield: 74.5%.

**Synthesis of Co*_x_*M*_(1-x)_*(H_2_O)_2_[Ni(CN)_4_]·4H_2_O (Co*_x_*M*_(1-x)_*-H_2_O; M = Mn, Fe) and Co*_x_*M*_(1-x)_*[Ni(CN)_4_] (Co*_x_*M*_(1-x)_*)**

A solution of a mixture of CoCl_2_·6H_2_O and MnCl_2_·4H_2_O for M’ = Mn (or Fe(NH_4_)_2_(SO_4_)_2_·6H_2_O for M’ = Fe) in given molar percentage (10 %, 25 %, 50 %, 75 %, 90 %, 0.30 mmol in total) in H_2_O was added to aqueous solutions of K_2_[Ni(CN)_4_]·H_2_O (72.29 mg, 0.30 mmol) and stirred for 24 h, washed with water, and dried in air. Dehydrated powder samples of **MNi** and **Co*_x_*M*_(1-x)_*** were prepared by heating **MNi-H_2_O** and **Co*_x_*M*_(1-x)_*-H_2_O** under vacuum at 353 K for 3 h.

**Physical Measurements**

**SEM-energy dispersive X-ray spectrometry (SEM-EDX)**

SEM-EDX images were measured using a Quanta^TM^ 250 FEG (FEI) instrument (electron high tension (EHT) value: 20.00 keV) at RT. To suppress the charging effect, the insulating powder samples were thinly expanded on the carbon tape. X-ray fluorescence analysis (XRF) was performed using a Shimadzu Rayny EDX-720 (X-ray voltage: 50 kV, X-ray current: 30 µA).

***In situ* Powder X-ray diffraction and Le Bail refinement**

Powder samples of **MNi-H_2_O** and **Co*_x_*M*_(1-x)_*-H_2_O** were sealed in soda glass capillaries with an inner diameter of 0.5 mm. *In situ* PXRD measurements for **MNi** and **Co*_x_*M*_(1-x)_*** were conducted under vacuum with a soda glass capillary connected to a gas-handling system (BELSORP-MAX, Microtrac BEL Corp.). The PXRD patterns of **MNi-H_2_O** and **Co*_x_*M*_(1-x)_*-H_2_O** were indexed using the FOX software,^3^ providing reasonable space group (*Pnma*) and cell parameters at RT with the same orthorhombic system as that of **MNi-H_2_O**. The Le Bail refinements were carried out using the RIETAN-FP software.^4^

**Water adsorption measurement**

Water adsorption and desorption isotherms were acquired using a BELSORP-MAX volumetric adsorption equipment (Microtrac BEL Corp.) at 298 K. The samples were dehydrated by heating them at 353 K for 6 h before the measurements.

***L*_2,3_-edge XAFS measurement**

*L*_2,3_-edge X-ray absorption fine structure (XAFS) measurements were performed at the BL-11 of the Synchrotron Radiation Center at Ritsumeikan University. The *L*_2,3_-edge XAFS spectra were obtained at RT in the partial electron yield (PEY) mode. A microchannel plate (MCP) for detecting the Auger and secondary electrons was set at an angle of 45° below the direction of photon propagation. To suppress the strong background caused by the C, N, and O *K*-edge absorptions in the spectra, a voltage of −550 V was applied to the gold mesh installed in front of the MCP. The sample preparation was performed in an Ar-filled glovebox to prevent H_2_O adsorption of atmospheric water; the sample was then transported to a vacuum chamber by using a transfer vessel without exposure to air. The photon energy for each sample was calibrated relative to the top of the *L*_3_-edge peak of Mn_2_O_3_ (641.5 eV), α-Fe_2_O_3_ (709.7 eV), and LiCoO_2_ (780.3 eV), respectively.

***K*-edge XAFS measurement**

*K*-edge absorption near-edge structure (XANES) and extended X-ray absorption fine structure (EXAFS) measurements were performed at the BL-15 of the SAGA Light Source. The XANES and EXAFS spectra were obtained in the transmission mode at RT by using two ionization chambers. The absorbance (−log(*I*_1_/*I*_0_)) was calculated from the incident X-ray intensity (*I*_0_) and the transmitted X-ray intensity (*I*_1_). A mixture of He 50%/N_2_ 50% and Ar 15%/N_2_ 85% was used as the ionization gases for *I*_0_ and *I*_1_, respectively. The photon energy was calibrated using a 3d transition metal foil at RT. The samples were diluted with high-purity boron nitride to an appropriate concentration for the XAFS measurement and molded into pellets in an Ar-filled glovebox. The pellet samples were then sealed in a polyethylene bags. Fourier-transform spectra were obtained using the *k*^2^-weighted EXAFS spectra, and the *K*-edge XAFS data were processed using the Athena and Artemis software.^5^

**Additional measurement**

IR spectra were recorded with a Perkin Elmer Spectrum with an ATR accessory in the range of 650–4000 cm^–1^.

**Fig. S1**. SEM images and elemental mapping of (a) **Co*_x_*Mn*_(1-x)_*-H_2_O** and (b) **Co*_x_*Fe*_(1-x)_*-H_2_O**

**Table S1.** Co Contents in **Co*_x_*Mn*_(1-x)_*-H_2_O** from XRF measurement

| Co ratio at preparation^a^ | 0.1 | 0.25 | 0.5 | 0.75 | 0.9 |
| --- | --- | --- | --- | --- | --- |
| Found Co contents (*x*)^b^ | 0.17 | 0.29 | 0.6 | 0.79 | 0.88 |
| Error^c^ | 2.43× 10^-3^ | 1.03× 10^-3^ | 1.62× 10^-3^ | 6.70× 10^-4^ | 3.27× 10^-4^ |

*^a^* The mixing ratio of Co ions to synthesize **Co*_x_*Mn*_(1-x)_*-H_2_O**.

*^b^* The contents of Co ions in each **Co*_x_*Mn*_(1-x)_*-H_2_O**.

*^c^* The Error was calculated from the 3 location-dependent measurements.

**Table S2.** Co Contents in **Co*_x_*Fe*_(1-x)_*-H_2_O** from XRF measurement

| Co ratio at preparation^a^ | 0.1 | 0.25 | 0.5 | 0.75 | 0.9 |
| --- | --- | --- | --- | --- | --- |
| Found Co contents (*x*)^b^ | 0.10 | 0.28 | 0.55 | 0.82 | 0.93 |
| Error^c^ | 1.55× 10^-3^ | 3.26× 10^-4^ | 6.62× 10^-4^ | 5.67× 10^-4^ | 4.02× 10^-4^ |

*^a^* The mixing ratio of Co ions to synthesize **Co*_x_*Fe*_(1-x)_*-H_2_O**.

*^b^* The contents of Co ions in each **Co*_x_*Fe*_(1-x)_*-H_2_O**.

*^c^* The Error was calculated from the 3 location-dependent measurements.

**Fig. S2**. PXRD patterns of (a) **Co*_x_*Mn*_(1-x)_*-H_2_O** and (b) **Co*_x_*Fe*_(1-x)_*-H_2_O,** and a simulated pattern of **CoNi-H_2_O** (black).^1,2^

**Fig. S3**. Le Bail analyses of **MNi-H_2_O** (M = Mn, Fe, and Co) with the results of PXRD patterns (*λ* = 1.5418 Å). Red dots, black line, and blue line represent the observed plots, calculated pattern, and their difference, respectively. Green bars are the calculated positions of the Bragg reflections.

**Fig. S4**. Le Bail analyses of **Co*_x_*Mn*_(1-x)_*-H_2_O** with the results of PXRD patterns (*λ* = 1.5418 Å). Red dots, black line, and blue line represent the observed plots, calculated pattern, and their difference, respectively. Green bars are the calculated positions of the Bragg reflections.

**Fig. S5**. Le Bail analyses of **Co*_x_*Fe*_(1-x)_*-H_2_O** with the results of PXRD patterns (*λ* = 1.5418 Å). Red dots, black line, and blue line represent the observed plots, calculated pattern, and their difference, respectively. Green bars are the calculated positions of the Bragg reflections.

**Table S3.** Refined cell parameters of **Co*_x_*Mn*_(1-x)_*-H_2_O** by using PXRD patterns shown in **Fig. S3 and S4**. The cell parameters are obtained by the Le Bail analyses using FOX and RIETAN-FP program.^3,4^

| *x* | 0 | 0.17 | 0.29 | 0.6 | 0.79 | 0.88 | 1 |
| --- | --- | --- | --- | --- | --- | --- | --- |
| Crystal system | Orthorhombic | Orthorhombic | Orthorhombic | Orthorhombic | Orthorhombic | Orthorhombic | Orthorhombic |
| Space group | *Pnma* | *Pnma* | *Pnma* | *Pnma* | *Pnma* | *Pnma* | *Pnma* |
| *a* / Å | 12.4401(2) | 12.3883(2) | 12.23660(5) | 12.3153(5) | 12.2573(5) | 12.2020(5) | 12.2014(2) |
| *b* / Å | 14.2793(4) | 14.2015(5) | 14.178(1) | 14.0763(8) | 13.9986(7) | 13.9337(7) | 13.9201(4) |
| *c* / Å | 7.3897(2) | 7.3387(2) | 7.3041(5) | 7.2615(4) | 7.2069(3) | 7.1711(3) | 7.1617(2) |
| *V* / Å^3^ | 1312.67(5) | 1291.11(7) | 1280.7(1) | 1258.8(1) | 1236.6(1) | 1219.2(1) | 1216.37(4) |
| *R_wp_* | 0.01733 | 0.00900 | 0.01419 | 0.00833 | 0.00746 | 0.00741 | 0.00694 |

**Table S4.** Refined cell parameters of **Co*_x_*Fe*_(1-x)_*-H_2_O** by using PXRD patterns shown in **Fig. S3 and S5**. The cell parameters are obtained by the Le Bail analyses using FOX and RIETAN-FP program.^3,4^

| *x* | 0 | 0.1 | 0.28 | 0.55 | 0.82 | 0.93 | 1 |
| --- | --- | --- | --- | --- | --- | --- | --- |
| Crystal system | Orthorhombic | Orthorhombic | Orthorhombic | Orthorhombic | Orthorhombic | Orthorhombic | Orthorhombic |
| Space group | *Pnma* | *Pnma* | *Pnma* | *Pnma* | *Pnma* | *Pnma* | *Pnma* |
| *a* / Å | 12.2078(4) | 12.1901(1) | 12.1932(2) | 12.2023(2) | 12.2108(2) | 12.2095(2) | 12.2014(2) |
| *b* / Å | 14.0212(8) | 14.0013(3) | 13.9910(3) | 13.9734(4) | 13.9492(3) | 13.9314(4) | 13.9201(4) |
| *c* / Å | 7.2239(3) | 7.2173(2) | 7.2076(2) | 7.1925(2) | 7.1789(2) | 7.1695(2) | 7.1617(2) |
| *V* / Å^3^ | 1236.5(1) | 1231.83(4) | 1229.58(4) | 1226.37(6) | 7.1789(2) | 1219.50(5) | 1216.37(4) |
| *R_wp_* | 0.01617 | 0.00709 | 0.00748 | 0.00665 | 0.00614 | 0.00627 | 0.00694 |

**Fig. S6**. IR spectra of (a) **Co*_x_*Mn*_(1-x)_*-H_2_O** and (b) **Co*_x_*Fe*_(1-x)_*-H_2_O**

**Fig. S7**. PXRD patterns of **MNi-H_2_O** (M = Mn, Fe, and Co) (black), **MNi** (red), and **MNi-H_2_O** (rehydration; blue).^2^

**Fig. S8**. PXRD patterns of **Co*_x_*Mn*_(1-x)_*-H_2_O** (black), **Co*_x_*Mn*_(1-x)_*** (red), and **Co*_x_*Mn*_(1-x)_*-H_2_O** (rehydration; blue)

**Fig. S9**. PXRD patterns of **Co*_x_*Fe*_(1-x)_*-H_2_O** (black), **Co*_x_*Fe*_(1-x)_*** (red), and **Co*_x_*Fe*_(1-x)_*-H_2_O** (rehydration; blue)

**Fig. S10**. TGA curves of **MNi-H_2_O** (M = Mn, Fe, Co, Ni).^2^

**Fig. S11**. *L*_2,3_-edge XAFS spectra of (a) **CoNi** (black), YBaCo_3_AlO_7_ (red), CoO (blue solid line), and Sr_2_CoO_3_Cl (blue dashed line). (b) **FeNi** (black) and CsFe_2_Se_3_ (red). (c) **MnNi** (black) and (Zn,Mn)Se (red).^2^

**Fig. S12**. Co *K*-edge XAFS spectra of (a) **Co*_x_*Mn*_(1-x)_*** and (b) **Co*_x_*Fe*_(1-x)_***. (b) Mn *K*-edge XAFS spectra of **Co*_x_*Mn*_(1-x)_***. (d) Fe K-edge XAFS spectra of **Co*_x_*Fe*_(1-x)_***.

**Fig. S13**. *K*-edge XANES spectra of **Co*_x_*M*_(1-x)_***. All spectra were recorded at room temperature in the transmittance mode.

**Table S5.** Distances between the metal ions (M) of **Co*_x_*Mn*_(1-x)_*** and the neighboring N (*d*_M-N_), C (*d*_M-C_), and Ni (*d*_M-N_i) atoms of the [Ni(CN)_4_]^2‒^ units

| Co contents (*x*)*^a^* | 0 | 0.17 | 0.29 | 0.6 | 0.79 | 0.88 | 1 |
| --- | --- | --- | --- | --- | --- | --- | --- |
| *d*_Co-N_ / Å | − | 1.5340 | 1.5647 | 1.5647 | 1.5647 | 1.5647 | 1.5647 |
| *d*_Mn-N_ / Å | 1.6874 | 1.6874 | 1.6567 | 1.6567 | 1.6567 | 1.6567 | − |
| *d*_Co-C_ / Å | − | 2.6078 | 2.5771 | 2.5771 | 2.5771 | 2.5771 | 2.5771 |
| *d*_Mn-C_ / Å | 2.6998 | 2.6998 | 2.6691 | 2.6691 | 2.6691 | 2.6691 | − |
| *d*_Co-Ni_ / Å | − | 4.5406 | 4.5406 | 4.5406 | 4.5406 | 4.5406 | 4.5099 |
| *d*_Mn-Ni_ / Å | 4.6326 | 4.6019 | 4.5713 | 4.5713 | 4.5713 | 4.5713 | − |

*^a^* The contents of Co ions in each **Co*_x_*Mn*_(1-x)_*-H_2_O** from XRF results.

**Table S6.** Distances between the metal ions (M) of **Co*_x_*Fe*_(1-x)_*** and the neighboring N (*d*_M-N_), C (*d*_M-C_), and Ni (*d*_M-N_i) atoms of the [Ni(CN)_4_]^2‒^ units

| Co contents (*x*)*^a^* | 0 | 0.1 | 0.28 | 0.55 | 0.82 | 0.93 | 1 |
| --- | --- | --- | --- | --- | --- | --- | --- |
| *d*_Co-N_ / Å | − | 1.5340 | 1.5647 | 1.5647 | 1.5647 | 1.5647 | 1.5647 |
| *d*_Fe-N_ / Å | 1.6260 | 1.6260 | 1.6260 | 1.6260 | 1.5953 | 1.6260 | − |
| *d*_Co-C_ / Å | − | 2.6384 | 2.5771 | 2.5771 | 2.5771 | 2.5771 | 2.5771 |
| *d*_Fe-C_ / Å | 2.6384 | 2.6078 | 2.6078 | 2.6078 | 2.6384 | 2.5771 | − |
| *d*_Co-Ni_ / Å | − | 4.5713 | 4.5406 | 4.5406 | 4.5406 | 4.5406 | 4.5099 |
| *d*_Fe-Ni_ / Å | 4.6019 | 4.5713 | 4.5713 | 4.5713 | 4.5406 | 4.5406 | − |

*^a^* The contents of Co ions in each **Co*_x_*Fe*_(1-x)_*-H_2_O** from XRF results.

**References in SI**

1 O. Reyes-Martinez, E. Torres-García, G. Rodríguez-Gattorno and E. Reguera, “Dehydration Process of Hofmann-Type Layered Solids,” *Materials* **2013**, *6*, 1452–1466.
2 H. Yoshino, K. Yamagami, H. Wadati, H. Yamagishi, H. Setoyama, S. Shimoda, A. Mishima, B. Le Ouay, R. Ohtani and M. Ohba, “Coordination Geometry Changes in Amorphous Cyanide-Bridged Metal–Organic Frameworks upon Water Adsorption,” *Inorg. Chem*. **2021**, *60*, 3338–3344.
3 V. Favre-Nicolin and R. Černý, “FOX, `free Objects for Crystallography’: A Modular Approach to Ab Initio Structure Determination from Powder Diffraction,” *J. Appl. Crystallogr*. **2002**, *35*, 734–743.
4 F. Izumi and K. Momma, “Three-Dimensional Visualization in Powder Diffraction. Solid State Phenomena,” *Solid State Phenom*. **2007**, *130*, 15–20.
5 B. Ravel and M. Newville, “ATHENA, ARTEMIS, HEPHAESTUS: Data Analysis for X-Ray Absorption Spectroscopy Using IFEFFIT,” *J. Synchrotron Radiat.* **2005**, *12*, 537–541.
